# Epithelial-Mesenchymal Transition during Metastasis of HPV-Negative Pharyngeal Squamous Cell Carcinoma

**DOI:** 10.1155/2018/7929104

**Published:** 2018-03-06

**Authors:** Friedrich Ihler, Ronja Gratz, Hendrik A. Wolff, Bernhard G. Weiss, Mattis Bertlich, Julia Kitz, Gabriela Salinas, Margret Rave-Fränk, Martin Canis

**Affiliations:** ^1^Department of Otorhinolaryngology, University Medical Center Göttingen, Georg-August-University Göttingen, Robert-Koch-Strasse 40, 37099 Göttingen, Germany; ^2^Department of Radiotherapy and Radiation Oncology, Pius-Hospital Oldenburg, Georgstrasse 12, 26121 Oldenburg, Germany; ^3^University Medical Center Göttingen, Georg-August-University Göttingen, Robert-Koch-Strasse 40, 37099 Göttingen, Germany; ^4^Strahlentherapie Radiologie München, Burgstrasse 7, 80331 München, Germany; ^5^Department of Pathology, University Medical Center Göttingen, Georg-August-University Göttingen, Göttingen, Germany; ^6^Transcriptome and Genome Analysis Laboratory (TAL), Department of Developmental Biochemistry, University Medical Center Göttingen, Georg-August-University Göttingen, Justus-von-Liebig-Weg 11, 37077 Göttingen, Germany; ^7^Department of Radiotherapy and Radiation Oncology, University Medical Center Göttingen, Georg-August-University Göttingen, Robert-Koch-Strasse 40, 37099 Göttingen, Germany

## Abstract

In epithelial tumors, a shift towards a mesenchymal phenotype has been associated with increased invasiveness and metastasis. It is assumed that this phenomenon plays a major role in disease progression and ultimately prognosis. This study investigated epithelial-mesenchymal transition (EMT) in human papillomavirus- (HPV-) negative pharyngeal squamous cell carcinoma. Tissue was obtained from one hypopharyngeal primary tumor and a regional lymph node metastasis during surgery with curative intention. A cell culture was established from the primary tumor and mesenchymal growth conditions were emulated. Gene expression profiling was performed (Human 8 × 60 K design array, Agilent Technologies) and EMT was assessed by a gene set (MSigDB: M5930, Hallmark_epithelial_mesenchymal_transition), applying gene set expression analysis (GSEA). Immunohistochemical staining and flow cytometry of CD44 and E-cadherin were compared in primary tumor, metastasis, and cell cultures. Primary tumor and metastasis were highly positive for CD44. A loss of E-cadherin occurred in the metastasis. Flow cytometry showed the appearance of a population without E-cadherin in spheroid colonies. In GSEA, the EMT phenotype was enriched in the primary tumor compared to metastasis and cell cultures (FDR < 25%, *p* < 5%). EMT showed variable expression during metastasis. It may thereby be a dynamic state in HPV-negative pharyngeal squamous cell carcinoma that is active only during the process of metastasis itself. Thereby, the primary tumor as well as the metastasis may exhibit fewer EMT properties.

## 1. Introduction

Head and neck squamous cell carcinoma (HNSCC) is a major tumor entity with a global share in cancer incidence of 3.8% for the oral cavity and pharyngeal sites [1]. Treatment options include surgery, radiotherapy, chemotherapy, and immunotherapy [2]. HNSCC consists of subtypes with specific properties. Pharyngeal squamous cell carcinoma negative for human papillomavirus (HPV), one such subtype, carries a particularly poor prognosis [3].

A shift to a mesenchymal phenotype in cancer cells has been identified as a major contribution to progression and metastasis of malignant epithelial tumors [4], including HNSCC [5]. This epithelial-mesenchymal transition (EMT) is originally a basic biological process in embryonic development and wound healing, whereas in a pathophysiological context it plays a role in organ fibrosis and cancer [6, 7]. EMT has been reported to increase invasiveness [6–8] and migration [6, 7], prevent apoptosis and senescence, and contribute to immunosuppression [6] and to the resistance to chemotherapy and immunotherapy [6, 9] in transformed cancer cells. It is mediated by the tumor microenvironment [10, 11] and leads to a loss of cell adhesion and epithelial markers, a gain of mesenchymal markers, and an alteration of the cytoskeletal structure towards a loss of cell polarity [6, 12, 13].

The loss of E-cadherin is widely seen as the hallmark for the completion of EMT in HNSCC as in other epithelial tumor entities [6, 8, 10, 13–15]. Expression of E-cadherin is tightly controlled through multiple signal transduction pathways and negatively regulated by transcription factors like Slug, Snail, and Twist [13, 16, 17]. E-cadherin is the main adhesion protein of epithelia and is responsible for cell attachment and epithelial polarity [18], while it is even considered as an inhibitor of EMT by some authors [19]. A loss of E-cadherin has independently been reported to be associated with poor prognosis [8, 20–22], tumor progression [10, 23], and metastatic spread [23].

A close relation between EMT and cancer stem cells (CSCs) has been described in many tumor entities [4, 20]. CSCs are tumorigenic cells capable of self-renewal and clonal generation of heterogeneous populations of cancer cells [24, 25]. Prince and coworkers characterized CSCs in HNSCC [26]. So far, no definite consensus exists on markers with optimal specificity for CSCs [10, 27]. Evidence points towards CD44 for the identification of CSCs in HNSCC [8, 26, 28] as in other tumor entities [10, 26, 27, 29, 30]. Clinically, CD44 is widely found in head and neck squamous cell carcinoma and is related to worse tumor characteristics and prognosis [31]. It is supposed to play an important role in tumor initiation [30], proliferation [28], and metastasis [26]. It has already been shown that overexpression of CD44 enhances cell proliferation and migration and increases cisplatin resistance and apoptosis inhibition in HNSCC cell lines [30]. On a cellular level, CD44-positive cells have been reported to contribute less than 10% to the total population of cancer cells [26] while other sources place the share of CD44-positive cells in HNSCC tumors in the range of 60–95% [32]. CD44 is constitutively expressed in permanent cell lines of HNSCC [33] and head and neck cancer cases were found to be CD44-positive in almost 60% [31]. The quantitative delineation of expression patterns is complicated by the existence of several isoforms of CD44 [28]. Therefore, the actual expression pattern of CD44 in HNSCC is not yet fully clear, but a predictive value on clinical properties relating to stemness has been reported for CD44 in HNSCC [8, 26, 28, 30, 31].

Most likely, a subset of CSCs shows an EMT phenotype [23], with EMT possibly representing the step from stationary to mobile cancer cells [34]. Evidence suggests that tumor cells capable of undergoing EMT may resemble CSCs and these cells may also be responsible for drug resistance and metastasis [14]. An alternative explanation describes EMT in cancer cells as a process crucial for the acquisition of stemness [35]. The frequent finding of metastases showing a greater degree of cellular differentiation than the corresponding primary tumor has been explained by EMT being a transient phenomenon with metastases undergoing a mesenchymal-epithelial transition (MET) at the target site [12, 13, 36]. Thereby, EMT and potentially stemness itself might be conditional, reversible, and interrelated dynamic states of cancer cells balanced by MET during and after metastasis [36, 37]. Moreover, it is even postulated that a tumor cell with EMT features has to transition back to an epithelial tumor phenotype to be able to accommodate and proliferate at a new organ site [13].

The specific role of EMT during metastasis in HNSCC is not yet fully elucidated. Therefore, the objective of this investigation was to characterize the expression of EMT markers during metastasis of a HPV-negative pharyngeal squamous cell carcinoma. The loss of E-cadherin was tracked as a hallmark of EMT development and the expression of CD44 as a marker of stemness. To that aim, a cell culture was established from a primary hypopharyngeal tumor and kept as spheroid colonies to emulate mesenchymal growth conditions. Furthermore, tumor tissue was investigated in comparison to a regional lymph node metastasis.

## 2. Results

### 2.1. Xenotransplantation Model and Establishment of Cell Cultures

**Figure 1:**
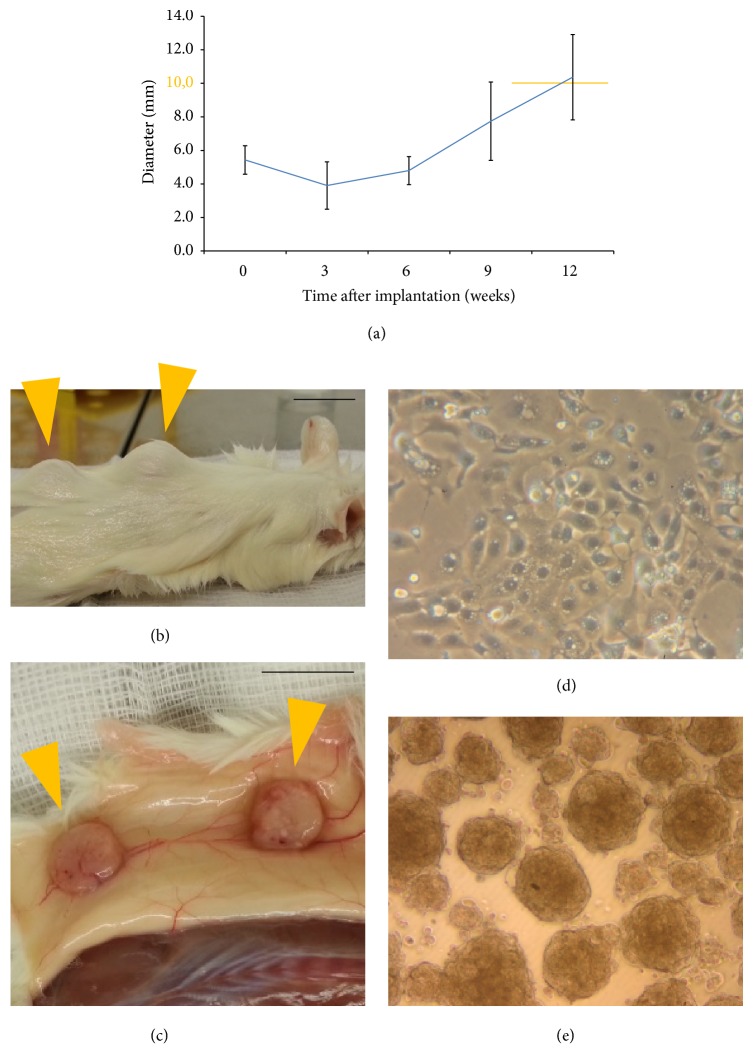
Growth characteristics of xenotransplants and cell cultures. (a) Growth rate of xenotransplants (10 samples); tumor tissue was harvested with a diameter of around 1 cm from week 10 on (highlighted in orange); (b) tumor growth visible from outside in NMRI mouse 73 days after xenotransplantation at 2 sites dorsally (orange arrows); (c) after euthanasia with macroscopically visible tumors located subcutaneously (orange arrows); scale bars: 1 cm. (d) Cell culture 56 days after dissociation (5x magnification); (e) spheroid colonies 6 days after transfer to ultralow attachment conditions (5x magnification).

Native samples from a hypopharyngeal squamous cell carcinoma were xenogenically implanted subcutaneously in the back of NMRI mice and showed growth from week 6 on in 50% of the sites (Figure 1(a)). Tumor tissue was harvested with a diameter of around 1 cm from week 10 on (Figures 1(b) and 1(c)). After dissociation of tumor tissue into a single cell suspension, cells could be propagated as primary cell culture (Figure 1(d)). Spheroid colony formation occurred 2–4 days after transfer of cells to ultralow attachment conditions (Figure 1(e)).

Tumor tissue was collected during initial surgical treatment of the 50-year-old male patient U. G. with a pT3 pN2b M0 HPV-negative squamous cell carcinoma of the dorsal wall of the hypopharynx. Initial treatment followed a curative intention. Therefore, the patient successfully received transoral laser microsurgery and bilateral selective neck dissection (R0). This was followed by postoperative chemoradiation of the region of the primary tumor, the regional lymphatic nodes, and the supraclavicular region with a total dose of 64.0 Gy and concomitant 40 mg/qm body surface area cisplatin weekly. After 25 months of follow-up, progressive osseous and pulmonary metastases were discovered without evidence of local or regional recurrence. The patient passed away 5 months later.

### 2.2. Tumor Stem Cell Properties

**Figure 2:**
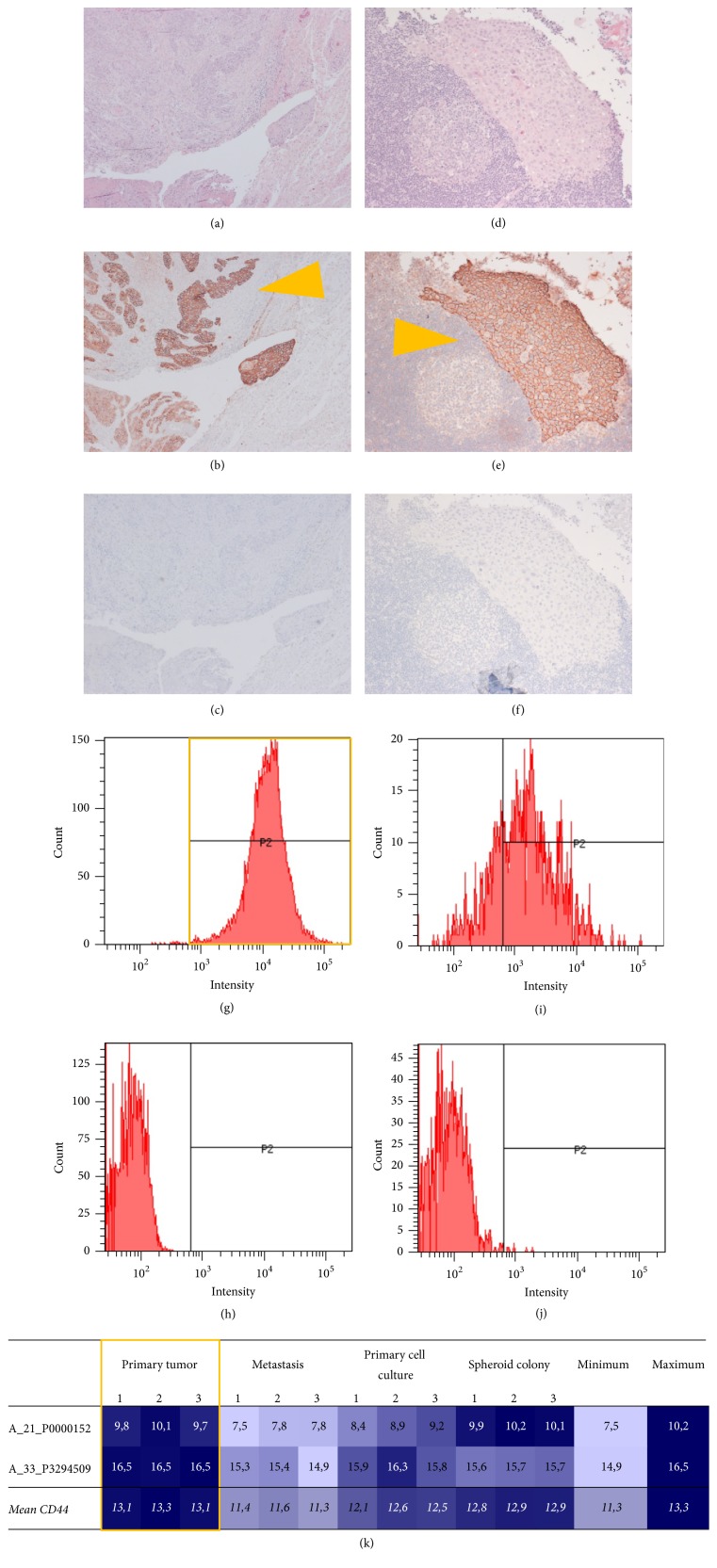
Expression of CD44 as stem cell marker. Immunohistochemistry showed expression of CD44 in both primary tumor and metastasis (orange arrows): (a–c) primary tumor, 10x magnification; (d–f) metastasis, 20x magnification; (a), (d) haematoxylin and eosin; (b), (e) haematoxylin and eosin and CD44; (c), (f) negative control. Flow cytometry showed high expression of CD44 in the primary cell culture (highlighted orange): (g–h) primary cell culture; (i–j) spheroid colony; (g), (i) CD44; (h), (j) negative control. (k) Transcriptome analysis showed the strongest signal of CD44 by the primary tumor (highlighted orange); columns: phenotypes/samples and minimum/maximum; rows: tags and mean values.

Histology of the tumor showed a moderately differentiated squamous cell carcinoma (G2) with partial signs of keratinization and extensive invasion of the subepithelial stroma (Figures 2(a) and 2(d)). CD44 was stained immunohistochemically as a marker of stemness and showed strong circular membranous staining in tissue from both primary tumor and metastasis (Figures 2(b), 2(c), 2(e), and 2(f)).

Comparable to a strong signal in tissue staining of the primary tumor, almost all cells from the primary cell culture (99.7%) were positive for CD44 in flow cytometry (Figures 2(g) and 2(h)). Cells from spheroid colonies showed a more heterogeneous picture, while still the majority of cells (54.7%) were positive for CD44 (Figures 2(i) and 2(j)).

Transcriptome analyses were used to quantify expression of CD44. Thereby, the primary tumor showed the strongest signal of CD44 with a maximum fold change (FC) of 1.29 (false discovery rate, FDR: 0.026%) compared to the metastasis (Figure 2(k)).

In summary, primary tumor tissue showed the strongest signs of stemness, followed by a cell culture derived directly from the primary tumor. CD44 was also expressed in samples from the metastasis, however in lower quantity corresponding to flow cytometry results of the spheroid colonies.

### 2.3. Epithelial Characteristics and Mesenchymal Shift

**Figure 3:**
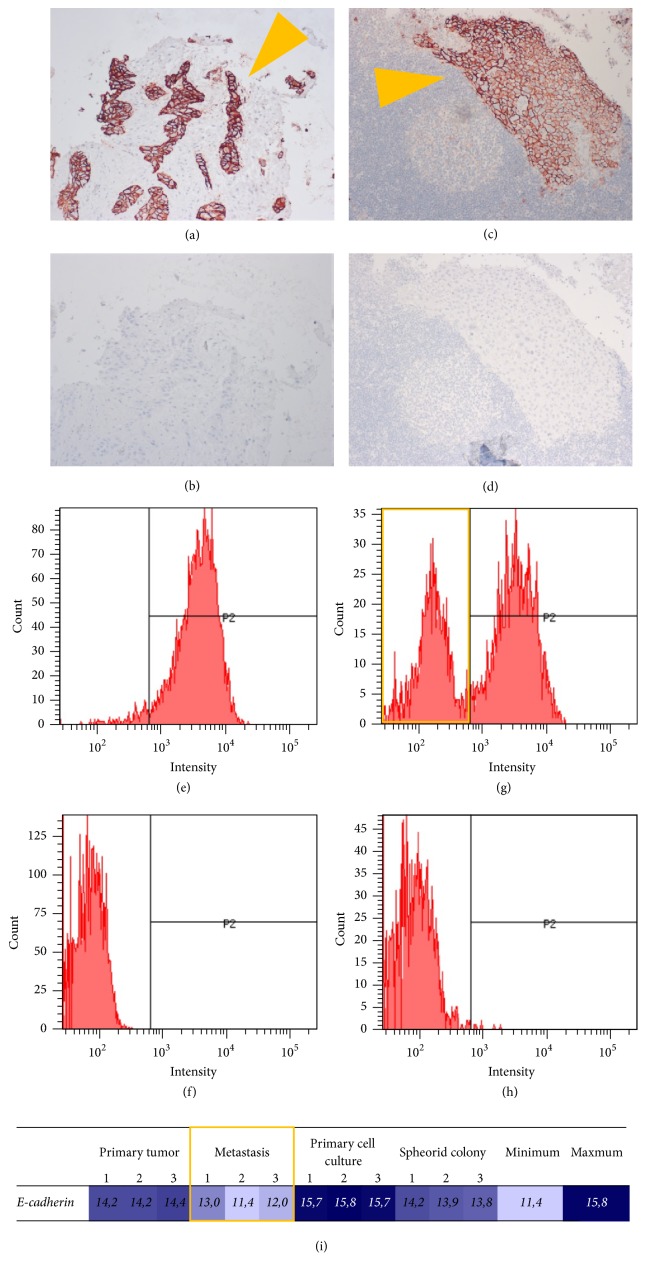
Expression of E-cadherin as epithelial marker. Immunohistochemistry showed expression of E-cadherin in primary tumor and metastasis (orange arrows): (a-b) primary tumor, 10x magnification; (c-d) metastasis, 20x magnification; (a), (c) haematoxylin and eosin and E-cadherin; (b), (d) negative control. Flow cytometry showed a population with E-cadherin-loss in the spheroid colony (highlighted orange): (e-f) primary cell culture; (g-h) spheroid colony; (e), (g) E-cadherin; (f), (h) negative control. (i) Transcriptome analysis showed the weakest signal for E-cadherin in the metastasis (highlighted orange); columns: phenotypes/samples and minimum/maximum; row: tag.

To delineate epithelial properties, the expression of E-cadherin was investigated. Samples from primary tumor and metastasis both showed a strong expression in immunohistochemistry staining (Figures 3(a) and 3(c)).

In flow cytometry, E-cadherin was highly expressed in the primary cell culture with almost all cells (96.3%) positive (Figures 3(e) and 3(f)). In spheroid colonies, besides E-cadherin positive cells (67.8%), a distinct E-cadherin-negative population appeared (Figures 3(g) and 3(h)), suggesting a shift in phenotype.

Transcriptome analysis showed particular differences between primary tumor and metastasis (FC 2.17, FDR 0.012%) as well as metastasis and primary cell culture (FC 3.61, FDR < 0.001%). Thereby, a significant loss of E-cadherin in the metastasis is found compared to the primary tumor and the cell culture derived from it. A loss of E-cadherin was less pronounced in spheroid colonies (Figure 3(i)).

Taken together, a loss of epithelial properties was encountered in samples from the metastasis. In spheroid colonies, a pronounced shift in phenotype was noted with sustained CD44-signal and simultaneous loss of E-cadherin.

### 2.4. EMT Signature

**Figure 4:**
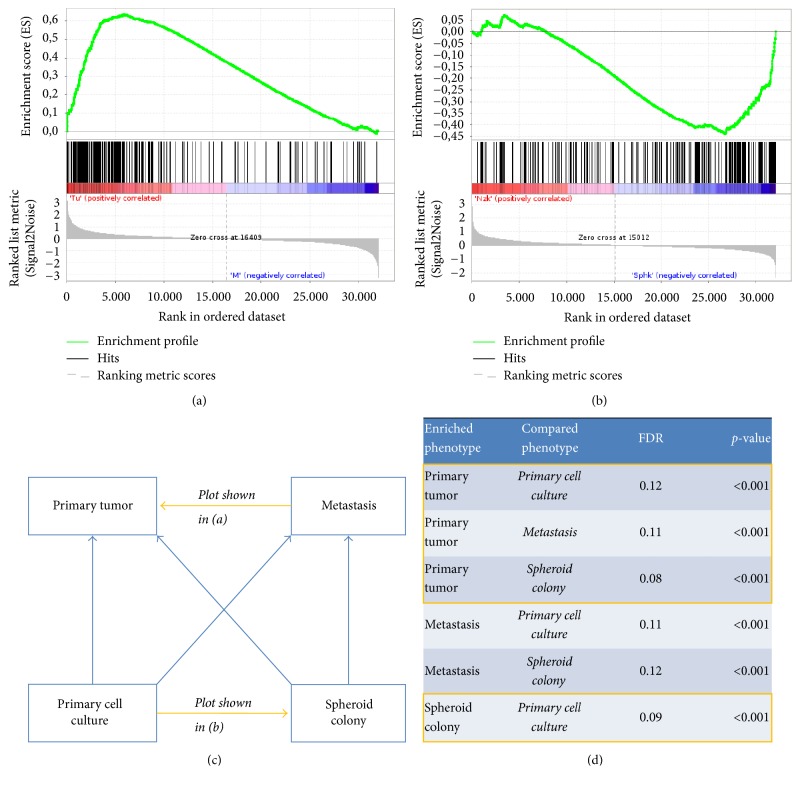
Gene set enrichment analysis of transcriptome data with a gene set representing epithelial-mesenchymal transition (Hallmark_epithelial_mesenchymal_transition, M5930). (a), (b) Enrichment plots: (a) enrichment of EMT in tumor over metastasis and (b) enrichment of EMT in spheroid colony over primary cell culture. (c), (d) Summary of enrichment results shows consistent enrichment of EMT in the primary tumor against all other phenotypes and enrichment in the spheroid colony against the primary cell culture (highlighted orange); (c) overview of phenotypes with direction of enrichment for EMT gene set in all pairwise comparisons; (d) false discovery rate (FDR) and *p* values.

By application of gene set enrichment analysis (GSEA), an EMT signature has been shown to be enriched in the primary tumor over the metastasis (Figure 4(a)). In cell culture phenotypes, the EMT gene set was enriched in the spheroid colony over the primary cell culture (Figure 4(b)), signifying successful enrichment of cells with EMT properties by ultralow attachment conditions. All pairwise comparisons showed a particularly strong statistically significant enrichment of the EMT gene set in the primary tumor compared to the other phenotypes. Secondarily, EMT was enriched in the metastasis against the cell culture (Figure 4(c)). Enrichment results all showed a false discovery rate (FDR) of < 25% and a *p* value of < 0.001 (Figure 4(d)).

## 3. Discussion

This study found evidence for a dynamic role of EMT in HPV-negative pharyngeal squamous cell carcinoma during the process of metastasis. Primary tumor tissue showed surprisingly high stemness properties compared particularly to a lymph node metastasis. Spheroid colonies obtained in ultralow attachment conditions were sufficient to enrich cells with an EMT signature. Epithelial properties were lost progressively from tumor and primary cell culture towards spheroid colonies and metastasis. A gene expression signature of EMT was also most highly enriched in primary tumor tissue. Those results suggest a highly active signal of stemness and EMT in the primary tumor that might explain clinically aggressive behavior. Stemness and EMT is partially turned back in the metastasis, while the epithelial properties of the original tissue are not recovered.

Limitations of this study include the fact that only tissue from one patient was investigated. However, the chosen approach allows the exemplary exploration of mechanisms that lead to the generation of new hypotheses for future investigations. That includes the study of solid tissue samples by flow cytometry to allow better quantification on a cellular level. A potential drawback of the ectopic xenograft approach applied here for tumor expansion is a potentially different selection of clones in the host animal compared to the original situation in the patient [38]. However, orthotopic xenograft and transgenic animal models can rarely be developed in HNSCC [38] and were therefore not judged as a viable alternative. We included a spheroid culture for the enrichment of a mesenchymal phenotype and for providing an additional intermediate model between conventional flat layer cell culture colonies and in vivo animal models as suggested before [39].

This study overcomes limits of several earlier reports investigating the process of metastasis in HNSCC. Most likely, HNSCC is a heterogeneous disease, where tumor development is supposedly driven by different mechanisms in distinct subtypes. A typical example for this assumption is HPV, a crucial factor that alters carcinogenesis [13]. Many studies so far did not determine or report on HPV status. Therefore, we explicitly focused on the investigation of a HPV-negative pharyngeal squamous cell carcinoma here.

A bioinformatical approach was chosen to achieve a comprehensive assessment of the EMT phenotype. Therefore, the software tool gene set enrichment analysis (GSEA) can be applied to explore concordant differences in the expression of gene sets between samples [40, 41]. Its initial application for functional analysis of gene expression data applies the hypothesis that weak but coordinated changes in functionally related genes can represent biologically meaningful findings [42, 43]. Here, GSEA has been employed to track the phenotypical shift by EMT, an alternative application that has already been described before [44, 45]. Earlier approaches applied the principles of gene set enrichment to uncover EMT in patient series with head and neck squamous cell carcinoma [46–48] and found the enrichment of EMT to be associated with either increased rate of recurrences [46, 47] or reduced survival [46, 48].

The successful generation of spheroids from fresh surgical specimen has already been reported before [49]. Here, a modified approach was chosen by heterotopic xenotransplantation of fresh tumor tissue and subsequent dissociation to overcome issues with microbiological contamination in the upper aerodigestive tract and fibroblast overgrowth known in de novo cell lines [38]. The appearance of spheroids 2–4 days after transfer of cells to ultralow attachment conditions here was even faster than what the reports from the literature would suggest [39]. The present spheroid colony is best described as tumorsphere with the hallmark of growth in a low attachment environment according to a recently proposed classification [39]. Many HNSCC cell lines have been reported to cluster into 3-dimensional spheroids [38], thereby making spheroid colonies a viable model. Spheroids in HNSCC are composed of an outer layer of proliferating cells, an inner layer of quiescent cells, and a core of necrotic cells, reflecting the growth pattern and spatial organization of a solid tumor [38]. It is supposed that each spheroid is derived from a single cell and represents clonal expansion [39]. Spheres are supposed to be more comparable to mesenchymal tissue in contrast to the polarity exhibited by epithelium. Thereby, the ability to survive and grow in attachment-free conditions has been linked to EMT [11, 35]. Consequently, spheroid colonies in ultralow attachment conditions were used primarily as a means to emulate mesenchymal growth conditions here. Alternatively, it is widely accepted that spheroid colonies are a way to enrich and expand CSCs in various tumor entities [39, 50] including HNSCC [51–53]. This makes the present approach suitable to study the interrelation between EMT and CSCs in the future.

A link between EMT and CSCs in HNSCC has already been reported earlier [14, 54]. In this tumor entity, EMT is important for metastasis and correlated with increased stem cell characteristics [20]. In an approach comparable to the present study, low attachment culture conditions led to the enrichment of cells with EMT characteristics in HNSCC cell lines that were suspected to be CSCs and showed enhanced colony forming ability and invasiveness [55]. This premise is carried further here to demonstrate that EMT and the stem cell marker CD44 are highly activated in the primary tumor of HPV-negative pharyngeal squamous cell carcinoma but are turned back in a metastasis.

An apparent question is whether an EMT itself confers stem cell properties upon cancer cells [6–8, 10] or a subset of CSCs is enabled by EMT to acquire additional features required for migration [11, 12, 20, 28, 56]. Evidence exists for the former hypothesis in HNSCC where CD44-positive cells showed increased migratory behavior and a more efficient tendency to form distant metastases compared to CD44-negative cells, a behavior typically linked to EMT [56]. However, this is not definite and at present it is not possible to draw a final conclusion. Pragmatically, CSCs and EMT may be seen as parallel events in the progression of carcinogenesis towards metastasis for the time being [4].

Beyond a close interrelation between EMT and CSCs, our findings suggest the existence of an at least partial revision of EMT after metastasis. This is illustrated by the surprisingly strong signal of CD44 and EMT in the primary tumor with considerably less expression in the metastasis. The notion that metastatic tumor cells may undergo a mesenchymal-epithelial transition (MET) to give rise to a metastasis at the target site has been suggested before [4, 36] and may explain why EMT is often not evident in metastases [12]. A possible explanation might be that for migrating cancer cells, differentiation signals at the target site are different from those in the primary tumor [6, 20, 57]. In HNSCC, it has already been demonstrated that some cells having undergone EMT are able to reverse this process [11]. It is therefore tempting to assume sequential steps of EMT and redifferentiation as the drivers of metastasis [5].

Potential therapeutic approaches arise from these assumptions. Patients with HNSCC might benefit from therapeutic strategies that inhibit EMT by blocking the crosstalk between tumor and stromal cells [10]. EMT is targeted in HNSCC cell lines by various agents, but side effects are a major issue since this is a basic biological process [14]. Inhibition of invasion in HNSCC cells might lead to reversion of EMT and could result in greater sensitivity to cytotoxic treatments [8]. So far, intra- and intertumoral heterogeneity limit the options for the development of targeted therapies [4] and should therefore be the direction of future research.

## 4. Conclusion

Taken together, this study found a strong signal of epithelial-mesenchymal transition (EMT) in the primary tumor of a HPV-negative pharyngeal squamous cell carcinoma which is turned back during the process of metastasis. The data supports the assumption that the concepts of cancer stem cells and EMT are interrelated and reversible mechanisms during metastasis.

Future investigations should identify markers and pathways for EMT beyond the ones investigated here. A deeper insight into the process of metastasis should then be applied to develop novel therapeutic approaches.

## 5. Methods

### 5.1. Ethics

This work has been carried out in compliance with the Declaration of Helsinki of the World Medical Association in the current form. For animal experiments, the EU Directive 2010/63/EU was observed. It was approved by the responsible Institutional Review Board (Lower Saxony State Office for Consumer Protection and Food Safety (LAVES), Postal Box  3949, 26029 Oldenburg, Germany) on February 1, 2010, with the reference number 33.14-42502-04-13/1105.

### 5.2. Tumor Tissue

Tissue from primary tumor and a regional lymph node metastasis was collected during initial surgical treatment at the first occurrence of the tumor. Tissue was transferred from operating theater to the laboratory within 20 minutes for immediate xenotransplantation and storage for subsequent analyses.

### 5.3. Xenotransplantation and Primary Cell Culture

Fresh tumor tissue was cut into pieces with a maximum diameter of 2 mm and implanted subcutaneously in anaesthetized NMRI-nu mice (RjOrl:NMRI-*Foxn1*^*nu*^/*Foxn1*^*nu*^, Janvier Labs SAS, Saint-Berthevin, France). After harvest from euthanized animals, tumor tissue was dissociated into a single cell suspension by the GentleMACS Dissociator (Miltenyi Biotec GmbH, Bergisch Gladbach, Germany) applying a standardized protocol. In brief, tissue was minced manually and subjected to repeated cycles of mechanical stirring and enzymatic digestion. The resulting suspension was filtered and centrifugalized. The pellet was gently resuspended in a medium (Airway Epithelial Cell Growth Medium with SupplementMix, PromoCell GmbH, Heidelberg, Germany) with the addition of 50 *μ*g/ml ampicillin (Ratiopharm GmbH, Ulm, Germany). Cells were incubated at 37°C in 5% CO_2_ for 4–7 days. Following that, the initial medium was gradually replaced by DMEM (Biowest, Nuaillé, France) with the addition of 10% fetal bovine serum, 14 *μ*g/ml phosphoethanolamine, 11 *μ*g/ml ethanolamine, 0.5 ng/ml EGF (all Biochrom AG, Berlin, Germany), 5.0 *μ*g/ml hydrocortisone, 5.0 *μ*g/ml insulin (Sigma-Aldrich, St. Louis, USA), 0.5 ng/ml FGF (R&D Systems Inc., Minneapolis, USA), and 50 *μ*g/ml ampicillin.

### 5.4. Spheroid Colony Formation

For the generation of spheroid colonies, a protocol analogous to earlier reports [52, 55] was developed. In brief, 4.0 × 10^4^ cells from the primary cell culture were transferred to ultralow attachment plates (Corning, New York, NY, USA) with serum-free DMEM/F-12 medium supplemented by N-2 and B-27 (Life Technologies, Carlsbad, USA) as well as 20 ng/ml human recombinants EGF and FGF (R&D Systems Inc., Minneapolis, USA).

### 5.5. Flow Cytometry

Analytic flow cytometry was performed with cells from the primary line or the spheroid colony by the BD FACSCanto II Flow Cytometer (BD Biosciences, San Jose, USA) with a wavelength of 488 nm up to a count of 10.000 events. For preparation, 1.25 × 10^5^ cells were incubated with either 5.0 *μ*l phycoerythrin-conjugated murine anti-human CD44 (BD Biosciences, Heidelberg, Germany), murine anti-human E-cadherin (BioLegend Inc., San Diego, USA) antibody, or PBS alone, centrifugalized, and resuspended.

### 5.6. Histology

Tumor tissue for immunohistochemistry was fixated in 4.5% formaldehyde solution (Merck KGaA, Darmstadt, Germany), dehydrated, and embedded in paraffin wax. Two micron sections were stained with hematoxylin and eosin (HE). The SAView (rabbit-HRP, AEC) IHC kit (Enzo Life Sciences, Farmingdale, USA) was applied for immunohistochemistry with antibodies to CD44 (rabbit, anti-human; Abcam plc, Cambridge, England) and E-cadherin (rabbit, anti-human; Cell Signaling Technology Inc., Danvers, USA), diluted 1 : 200. As negative control, antibody diluent was used alone.

### 5.7. mRNA Extraction and Transcriptome Analysis

Global gene expression analysis was performed by the Human 8 × 60 K design array (Agilent Technologies Inc., Santa Clara, USA). 200 ng of total RNA was used as starting material extracted from tumor tissue or 1.0 × 10^6^ cells. cDNA synthesis and in vitro transcription were performed according to the manufacturer's recommendation. Quantity and efficiency of the labeled amplified cDNA were determined using the NanoDrop 1000 Spectrophotometer version 3.2.1 (Thermo Fisher Scientific Inc., Waltham, USA).

The Agilent RNA Spike-In Kit for One Color (Agilent Technologies Inc., Santa Clara, USA) was applied for hybridization using a standardized protocol for 17 hours at 10 rpm and 65°C in the Microarray Hybridization Oven (Agilent Technologies Inc., Santa Clara, USA). Washing and staining of the arrays were performed according to the manufacturer's recommendation. Cy3 intensities were detected by one-color scanning using the Agilent G2505B DNA Microarray Scanner System (Agilent Technologies Inc., Santa Clara, USA) at a 3 micron resolution. Scanned image files were visually inspected for artifacts and then analyzed.

### 5.8. Microarray Data Analysis

In compliance with the MIAME Standard (http://fged.org/projects/miame) [58], the data discussed in this publication has been deposited in NCBI's Gene Expression Omnibus (GEO) [59] and is accessible through GEO series accession number GSE97510 (https://www.ncbi.nlm.nih.gov/geo/query/acc.cgi?acc=GSE97510).

Quantile normalization of microarray data was applied to the log2-transformed intensity values as a method for between-array normalization to ensure that the intensities had similar distributions across arrays. Data was analyzed by gene set enrichment analysis (GSEA) and developed by the Broad Institute, Cambridge, USA (http://broad.harvard.edu/gsea) [40, 41]. GSEA was performed with a computationally generated gene set representing genes involved in epithelial-mesenchymal transition (standard name: Hallmark_epithelial_mesenchymal_transition, systematic name: M5930) [60].
